# Mental wellbeing during the time of COVID-19 pandemic: A national pilot survey in Ethiopia

**Published:** 2022-04-09

**Authors:** Bethelhem Fekadu, Medhin Selamu, Eyerusalem Getachew, Hanna Negussie, Sewit Timothewos, Winini Belay, Abigiya Wondimagegnehu, Tigist Eshetu, Tigest Ajeme, Kehabtimer Shiferaw, Tsegahun Manyazewal, Abebaw Fekadu, Girmay Medhin, Charlotte Hanlon

**Affiliations:** 1 Addis Ababa University, Centre for Innovative Drug Development and Therapeutic Trials for Africa (CDT- Africa), Addis Ababa, Ethiopia; 2 Addis Ababa University, WHO Collaborating Center for Mental Health Research and Capacity Building, Department of Psychiatry, College of Health sciences, Addis Ababa, Ethiopia; 3 Addis Ababa University, College of Health Sciences, School of Public Health, Addis Ababa, Ethiopia; 4 Martin-Luther-University, Institute of Medical Epidemiology, Bio statistics and Informatics, Halle, Germany; 5 Brighton and Sussex Medical School, Global Health and Infection Department, Brighton, UK; 6 Addis Ababa University, Aklilu Lemma Institute of Pathobiology, Addis Ababa, Ethiopia.; 7 King’s College London, Institute of Psychiatry, Psychology and Neuroscience, Centre for Global Mental Health, London, UK

**Keywords:** COVID-19, mental wellbeing, mental health, disability, Ethiopia

## Abstract

**Introduction:**

The Coronavirus Disease 2019 (COVID-19) pandemic substantially disrupts population health and wellbeing globally, while little is known about the effect on mental wellbeing in developing countries. This study aimed to assess the impact of COVID-19 on mental wellbeing of individuals and households in Ethiopia.

**Methods:**

A cross-sectional, national pilot survey was conducted through phone interviews from September to November 2021. Mental wellbeing and disability were assessed using a questionnaire adapted from the 5-item World Health Organization Wellbeing Index (WHO-5), the Oslo Social Support Scale (OSSS-3), and the WHO Disability Assessment Scale (WHODAS 2.0).

**Results:**

A total of 614 adults completed the pilot survey. The mean age was 36 years (standard deviation 11) and 71.7% were male. Mental wellbeing was poor in 218 (35.5%) participants. The most important predictors for poor mental wellbeing were rural residence (Adjusted Odds Ratio [AOR] 1.89; 95% CI 1.14, 3.14; p=0.012), perceived COVID-19 risk (AOR 1.75; 95% CI 1.18, 2.60; p=0.005), household stress (AOR 2.09; 95% CI 1.31, 3.34; p=0.002), experience of symptom of COVID-19 in the household (AOR 2.14; 95% CI 1.13, 4.04; p=0.019), and poor social support (AOR 2.43; 95% CI 1.51, 3.91; p<0.001).

**Conclusion:**

The study provides evidence that COVID-19 had a significant adverse impact on the mental wellbeing of individuals and households in Ethiopia. Further studies are needed to understand in detail the implications of the pandemic and interventions needed to keep mental wellbing of citizens.

## Introduction

The Coronavirus Disease 2019 (COVID-19) pandemic has impacted the physical, social and mental wellbeing of people globally (1). The very limited initial knowledge and the nature of the spread of the pandemic had required drastic change and adaptation at the individual, community and societal level with consequent rise in the level of stress among individuals and communities (2).

The adaptations have included implementation of good hygiene practices, social distancing, quarantine measures and isolation, most of which are likely to expose people to continuous stress while disrupting the normal ways of living and social networks (3).

These mental wellbeing challenges are not peculiar to this pandemic; for example past public health epidemics, such as severe acute respiratory syndrome (SARS), have been associated with unfavorable effect on mental wellbeing (4–6). During COVID-19 decreased mental wellbeing and an increase in mental health problems were reported in some studies (7, 8). A meta-analysis done on population based studies during COVID-19 pandemic reported high prevalence of depression (33.7%), anxiety (31.9%), and stress (29.6%) (9). A study from a town in southern Ethiopia has also indicated high prevalence of mental distress during the COVID-19 lockdown: depression 37.7%, anxiety 39.0%, and stress symptoms as 44.2 % (10).

The COVID-19 pandemic continues to disrupt the life of individuals, communities and healthcare providers (11). The psychological effects of this disease might even be higher in Ethiopia and other low- and middle-income countries (LMICs) as a result of resource constraints, weaker health systems, including inadequately developed mental healthcare system although little studied. There is a need to develop population-level mental health interventions that are believed to be well-suited where there is a lack of human and material resources in the sector (12). The aim of this study was to assess the mental wellbeing of Ethiopians during the COVID-19 pandemic and determine factors associated with the mental wellbeing status.

## Methods

### Study Design and sampling

Details of the methods are described elsewhere in this issue (13) and will be presented here briefly. The study recruited participants from all nine regional states of Ethiopia and the two chartered cities (Addis Ababa and Dire Dawa). The study period was from September to November 2021. The study was a population-based national cross-sectional survey using mobile phone interviews. Adults aged 18 and above who were able to provide information about themselves and their family were invited to take part in this study. The study was conducted in the three main languages of Ethiopia: Amharic, Afan Oromo and Tigrigna.

From a sampling frame of 11 million people with mobile phones starting with 0910 to 0920, random samples of 30,000 were selected. For the pilot, the first 614 participants from this random sample who responded and agreed to participate were included. The study included all the study participants who agreed to participate during the pilot study data collection period which was one month. As the study was an initial pilot survey for a larger cohort study, formal sample size calculation was not conducted. The study was conducted across Ethiopia and all participants contacted were from parts of the country since study participants were randomly contacted.

### Assessments

Subjective mental wellbeing was the main outcome of interest.

In addition to subjective mental wellbeing: disability, relevant individual and household risk factors were assessed.

### Wellbeing

Subjective mental wellbeing was assessed using the 5-item World Health Organization Well-Being Index (WHO-5), a widely used ‘condition neutral’ tool (14) and validated in Ethiopia (15). The items are only positively phrased and include the following: (1) I have felt cheerful and in good spirit, (2) I have felt calm and relaxed, (3) ‘I have felt active and vigorous’, (4) ‘I woke up feeling fresh and rested’ and (5) ‘My daily life has been filled with things that interest me’.

The respondents were asked to rate how well each of the 5 statements applies to him/her in the past four weeks or the past 30 days. Each of the 5 items is scored from 5 (all of the time) to 0 (none of the time). The total raw score would therefore range from 0 (absence of well-being) to 25 (maximal well-being). Conventionally, health-related quality of life measures are converted to a percentage scale from 0 (absent) to 100 (maximum), it is recommended to multiply the raw score of the WHO-5 by 4 (14) to transform the raw scores of the WHO-5 into the more conventional score. We, therefore, multiplied the total score of each participant by four to obtain the recommended range of scores. When used as a screening tool, a score of <50 in the WHO-5 is considered indicative of compromised mental wellbeing and depression(16).

### Disability

Two items from the World Health Organization Disability Assessment Schedule (WHODAS) 2·0 scale were used. The questions focused on the past month and enquired (1) for how many days the participant was totally unable to carry out his/her usual activities or work because of any health condition; (2) Excluding the days that the person was totally unable to carry out his/her activities, for how many days he/she had to cut back or reduce their usual activities or work because of any health condition.

### Sociodemographic measures

Sociodemographic and economic data considered relevant for mental wellbeing at the participant and household level were assessed using simple structured questionnaire that consisted of basic characteristics such as age, marital status, residence, educational status, occupation and region as well as income status.

### Household and participant level risk factors

Risk factors included medical conditions in the household that may complicate the course of COVID-19such as chronic medical conditions (hypertension, heart disease, asthma, TB, liver disease, kidney disease, diabetes) which may require some life style modifications for the family; and household level conflict that may affect mental wellbeing and assessed by asking whether there is an increase in stress and conflict in the household for the past one month of data collection period. Social support was assessed at the participant and household level using the Oslo 3-items social support scale (OSS). In addition, participants were also asked their perceived risk for COVID-19 and if they have been experiencing COVID-19 like symptoms in the previous month.

### Data collection procedures

Data were collected through mobile phone interviews using an electronic data capture platform. Data collectors were trained on the instruments and ethical data collection. Whenever the phone number didn’t work or wasn’t answered on the first try, retries were made up to three times before being excluded. The data collectors took over the data collection work once all contracts and training were completed. The survey procedures and instruments were pre-tested with 50 interviews for benefit, feasibility and acceptance and adjusted on the basis of the results of the pre-test.

### Data analysis

Data was exported to Stata version 14 (StataCorp, 1985–2013) for statistical analysis. For the primary outcome variable (Wellbeing index 5), total score was generated by adding up the items for each scale. The result was multiplied by four with a total score extending to 100. As per the recommendation, a cut-off value of 50 was taken as a wellbeing threshold, with those scoring below 50 categorized as experiencing low or poor wellbeing.

Descriptive analysis was used to explore the socio demographic, personal and household level stress related factors that are believed to be linked with wellbeing score. Multivariate logistic regression was used to evaluate factors associated with poor mental wellbeing (i.e. sex, age, marital status, place of work, perceived social support, perceived household stress, and perceived COVID-19 risk).

## Results

### Socio-demographic characteristic of study participants

A total of 614 participants were included in the study. Of those, 440 (71.7%) were male, 213 (34.7%) were in the age group of 30–39 years and most (78%; n=478) lived in urban areas. The mean (±Standard Deviation; SD) age of the participants was 36 (SD 11) years. Most were self-employed (42.3%; n=260) or government employees (n=178; 29%). More than two thirds (68.2%; n=419) of participants were married (Table 1).

### Mental wellbeing, disability and social support during COVID-19

The mean (±SD) score of the WHO-5 wellbeing scale in all the sample was 60.08 (±27.9). Low wellbeing was reported by 35.5% (n=218) of participants.

In terms of disability or ability to function in the past 30 days, all in all, over half of the participants had some impairment for at least a day, with a third of participants (n=202; 32.9%) reporting total inability to carry out their usual activities at least for a day because of any health condition at the time of COVID-19. Participants who were forced to reduce or cut back their usual activity at least for one day for the past one month in the time of COVID-19 were slightly lower (n=174; 28.3%).

Over a quarter (n=173; 28.18%) reported poor social support, with the rest reporting strong (n=220; 35.83%)) and intermediate social support (n=221; 36%). Significantly higher proportion of those with poor social support had compromised or poor mental wellbeing (*X*^2^=17.97; P<0.001).

### Factors associated with mental wellbeing during the COVID-19 pandemic

In the multivariable logistic regression model, poor mental wellbeing was significantly higher among those who reported an increase in household stress and conflict during the pandemic (AOR 2.09; 95% CI 1.31, 3.34), those who perceived that they were at risk of COVID-19 (AOR 1.75; 95% CI 1.18, 2.60), had someone with a chronic illness in the household (AOR 1.72; 95% CI 1.12, 2.64) or they had a symptom of COVID-19 in the past month (AOR 2.14; 95% CI 1.13, 4.04). Compared to those with good social support, the odds of poor mental wellbeing was increased in those with intermediate (AOR 1.61; 95% CI 1.03, 2.49) and poor social support (AOR 2.43; 95% CI 1.51, 3.91). Rural residence was also independently associated with poor mental wellbeing (AOR 1.89; 95% CI 1.14, 3.14). See table 2.

## DISCUSSION

There is consistent evidence from the literature of high income countries and some low and middle income countries that COVID-19 affects mental health negatively (17). Although selection bias, for example, those likely to have some anxiety may be more likely to volunteer for interview, could affect the quality and reliability of data in this study, a larger study from a cohort sample has indicated an increase in mental illness during COVID-19. This British cohort study found that mental distress rose from 18.9% during the pre-pandemic time to 27.3% during the early lock down of COVID-19. Similarly, the Global Health Questionnaire score rose from 11.5 during the pre-pandemic time to 12.6 in the early lock down periods of the pandemic (18).

Hence, COVID-19 is a likely explanation of the high level of poor mental wellbeing in our study. This is supported further by the association of poor mental wellbeing with concerns of contracting COVID-19 and living with someone who might have complicated course of illness if they contracted the illness. Again the association with low levels of social support engendered by the required life style changes during the COVID-19 era may partly explain the increase in poor mental wellbeing.

Nevertheless, a larger scale prospective study is required to have a clearer picture of the ongoing impacts of the COVID-19 pandemic. As part of such a study, any maladaptive behaviors, such as increase in alcohol consumption, need to be evaluated.

Interventions to mitigate the social disruptions caused by the disease and the public health control measures are also required. Such measures need to be locally developed or adapted and scalable.

The association of rural residence with poor mental wellbeing is in line with a previous population based study conducted prior to the pandemic (19). Understanding the vulnerabilities and risk factors among the rural population and developing community level mental wellbeing promotion interventions to tackle such risk factors need proper attention (12).

## Conclusion

This is the first national evaluation of the impact of COVID-19 pandemic on mental wellbeing of Ethiopians. Although selection bias is an important concern, the study has found poor mental wellbeing in over a third of participants, which is a relatively high rate. Further large scale cohort studies are needed to understand the impact of COVID-19 and to evaluate the consistency of the risk factors that need to be considered in any intervention plan. Locally developed or adapted interventions may also need to be prioritized.

## Figures and Tables

**Table 1:** Socio-demographic characteristics in relation to mental wellbeing score

Characteristics	Response Category	Total (%)	Mental Wellbeing Score ≤ 50		X^2^	P Value
			Number (%)			
			Yes	No		
SEX	Male	440 (71.7)	156 (34.5)	84 (64.6)	0.00	0.967
	Female	174 (28.3)	62 (35.6)	112 (64.2)		
AGE	18–29 years	207 (33.7)	79 (38.2)	128 (61.8)	1.58	0.663
	30–39 years	213 (34.7)	76 (35.7)	137 (64.3)		
	40–49 years	118 (19.2)	37 (31.4)	81 (68.6)		
	≥50 Years	76 (12.4)	26 (34.2)	50 (65.8)		
RESIDENCE	Urban	478(77.9)	159 (33.3)	319 (66.7)	4.73	0.030
	Rural	136 (22.2)	59 (43.4)	77 (56.6)		
REGION	Addis Ababa	222 (36.2)	71 (32.0)	151 (68.0)	5.33	0.255
	Oromia	144 (23.5)	53 (36.8)	91 (63.2)		
	Amhara	139 (22.6)	59 (42.5)	80 (57.6)		
	SNNPR	66 (10.8)	23 (34.9)	43 (65.2)		
	Others	43 (7.0)	12 (27.9)	31 (72.1)		
LEVEL OF EDUCATION	Primary school	55 (9.0)	17 (30.9)	38 (69.1)	2.72	0.435
	Secondary school	124(20.2)	40 (32.3)	84 (67.7)		
	Certificate	148 (24.1)	60 (40.5)	88 (59.5)		
	College/University	287 (46.7)	101 (35.2)	186 (64.8)		
OCCUPATION	Farmer/Pastoralist	56(9.1)	19 (33.9)	37 (66.1)	5.76	0.330
	Self-employed	260(42.4)	92 (35.4)	168 (64.6)		
	Government employee	178 (29.0)	63 (35.4)	115 (64.6)		
	Housewife	30 (4.9)	6 (20.0)	24 (80.0)		
	Unemployed	45 (7.3)	17 (37.8)	28 (62.2)		
	Others	45 (7.3)	21 (46.7)	24 (53.3)		
MARITAL STATUS	Single	172(28.0)	65 (37.8)	107 (62.2)	0.54	0.761
	Married	419 (68.2)	145 (34.6)	274 (65.4)		
	Divorced/widowed	23 (3.8)	8 (34.8)	15 (65.2)		
RELATIVE WEALTH	Very low	53 (8.6)	19 (35.9)	34 (64.2)	7.47	0.024
	Low	225(36.6)	95 (42.2)	130 (57.8)		
	Average and above	336 (54.7)	104 (31.0)	232 (69.1)		
LIVING WITH PEOPLE AGED ≥65	No	491(79.9)	167(34.01)	324(65.9)	2.38	0.123
	Yes	123(20.1)	51(41.46)	72(58.54)		
HOUSEHOLD CO MORBIDITY	No	466(75.9)	151(32.4)	315(67.6)	8.12	0.004
	Yes	148(24.1)	67(45.3)	81(54.7)		
PERCEIVED RISK FOR COVID 19	No	317(51.6)	92(29.1)	225(70.9)	12.02	0.001
	Yes	297(48.3)	126(42.4)	171(57.6)		
HOUSEHOLD SYMPTOM PAST ONE MONTH	No	562(91.5)	193(34.3)	369(65.7)	3.92	0.048
	Yes	52(8.4)	25(48.1)	27(51.9)		
PERCEIVED INCREASE IN STRESS AND CONFLICT IN HOUSEHOLD	No	509(82.9)	163(32.1)	346(67.9)	15.75	
	Yes	105(17.1)	55(52.4)	50(47.6)		<0.001
SOCIAL SUPPORT	Strong support	220(35.8)	161(40.7)	59(27.1)	17.97	
	Intermediate support	221(36)	144(36.36)	77(35.3)		<0.001
	Poor support	173(28.2)	91(22.9)	82(37.6)		

**Table 2:** Factors associated with poor mental wellbeing during the COVID-19 pandemic in Ethiopia

Characteristics	Response Category	Crude Odds Ratio (95% Confidence Interval)	Adjusted Odds Ratio (95% Confidence Interval)	P Value
Sex	Male	Ref	Ref	
	Female	1.01 (0.69, 1.45)	1.36 (0.87, 2.12)	0.169
Age (years)	18–29	Ref	Ref	
	30–39	0.89 (0.60, 1.33)	1.03 (0.64, 1.67)	0.877
	40–49	0.74 (0.45, 1.19)	0.83 (0.46, 1.50)	0.544
	50 and above	0.84 (0.48, 1.46)	1.01 (0.50, 2.00)	0.990
Residence	Urban	Ref	Ref	
	Rural	1.53 (1.04, 2.26)	1.89 (1.14, 3.14)	0.013
Region	Addis Ababa	Ref	Ref	
	Oromia	1.23 (0.79, 1.92)	1.02 (0.61, 1.69)	0.922
	Amhara	1.56 (1.01, 2.43)	1.29 (0.76, 2.19)	0.340
	SNNPR	1.13 (0.63, 2.03)	0.77 (0.39, 1.53)	0.471
	Others	0.82 (0.39, 1.69)	0.52 (0.22, 1.20)	0.128
Occupation	Farmer/pastoralist	Ref	Ref	
	Self-employed/daily labourer	1.06 (0.58, 1.96)	0.98 (0.43, 2.22)	0.975
	Government employee	1.06 (0.56, 2.01)	1.01 (0.42, 2.41)	0.985
	Housewife	0.48 (0.17, 1.39)	0.61 (0.18, 2.11)	0.444
	Unemployed	1.18 (0.52, 2.67)	1.06 (0.38, 2.95)	0.900
	Others	1.70 (0.76, 3.81)	1.84 (0.66, 5.07)	0.238
Level of education	Primary school	Ref	Ref	
	Secondary school	1.06 (0.53, 2.11)	1.30(0.61, 2.79)	0.494
	Certificate	1.52 (0.78, 2.94)	1.78(0.82, 3.87)	0.144
	College/University	1.21 (0.65, 2.25)	1.56(0.71, 3.44)	0.266
Marital status	Single	Ref	Ref	
	Married	0.87 (0.60, 1.25)	0.96(0.61, 1.53)	0.883
	Divorced/widowed	0.87 (0.35, 2.18)	1.02(0.36, 2.91)	0.958
Relative wealth	Very low	Ref	Ref	
	Low	1.31 (0.70, 2.43)	1.39(0.70, 2.75)	0.343
	Average and above	0.80 (0.43, 1.47)	0.89(0.45, 1.75)	0.737
Living with people aged ≥65	No	Ref	Ref	
	Yes	1.37(0.91, 2.05)	1.32(0.82, 2.12	0.242
Household stress	No	Ref	Ref	
	Yes	2.33 (1.52, 3.57)	2.09 (1.31, 3.34)	0.002
Perceived covid-19 risk	No	Ref	Ref	
	Yes	1.80 (1.28, 2.51)	1.75 (1.18, 2.60)	0.005
Household co-morbidity	No	Ref	Ref	
	Yes	1.72 (1.18, 2.51)	1.72 (1.12, 2.64)	0.012
Household symptom past 1 month	No	Ref	Ref	
	Yes	1.7 (0.99, 3.13)	2.14 (1.13, 4.04)	0.019
Social support	Strong support	Ref	Ref	
	Intermediate support	1.45 (0.97, 2.19)	1.61 (1.03, 2.49)	0.034
	Poor support	2.41 (1.54, 3.79)	2.43 (1.51, 3.91)	<0.001

## Data Availability

The datasets supporting the conclusions of this article are included within the article and its additional files. Any additional material can be obtained upon reasonable request.

## Abbrevations

COVID 19: Coronavirus Disease 2019
LMICs: low and middle income countries
SARS: Severe acute respiratory syndrome
WHO-5: 5 -item World Health Organization Well-Being Index
